# Controlled Crystallization Enables Facile Fine-Tuning of Physical–Chemical Properties of Nicergoline Toward Easier Processability

**DOI:** 10.3390/ph18101465

**Published:** 2025-09-29

**Authors:** Barbora Blahová Prudilová, Roman Gabriel, Michal Otyepka, Eva Otyepková

**Affiliations:** 1 Department of Physical Chemistry, Faculty of Science, Palacký University Olomouc, 17. Listopadu 12, 771 46 Olomouc, Czech Republic; barbora.blahovaprudilova@upol.cz (B.B.P.); roman.gabriel@tapi.com (R.G.); 2 Regional Centre of Advanced Technologies and Materials, Czech Advanced Technology and Research Institute (CATRIN), Palacký University Olomouc, Šlechtitelů 27, 783 71 Olomouc, Czech Republic; 3 TAPI Czech Industries s.r.o., Research & Development, Ostravská 29, 747 70 Opava, Czech Republic; 4 IT4Innovations, VSB—Technical University of Ostrava, 17. Listopadu 2172/15, 708 00 Ostrava-Poruba, Czech Republic

**Keywords:** active pharmaceutical ingredient (API), nucleation, seeding, controlled crystallization, sonocrystallization

## Abstract

**Background/Objectives**: Crystallization is a key process in the manufacturing of active pharmaceutical ingredients (APIs), as it significantly affects the physical and chemical properties of the final product. Nicergoline, a clinically relevant ergot derivative, was chosen as a model compound to investigate how different crystallization strategies affect particle attributes. The objective of this study was to compare controlled and uncontrolled crystallization techniques and evaluate their impact on the physicochemical properties of nicergoline. **Methods**: Nicergoline was crystallized using controlled methods, including sonication-induced and seeding-induced crystallization, and uncontrolled methods, namely cubic and linear cooling, as well as acetone evaporation. The resulting powders were characterized by using a range of physicochemical techniques to assess particle morphology, size distribution, agglomeration behavior, and surface properties. **Results**: Uncontrolled crystallization methods produced particles prone to agglomeration, resulting in a broader particle size distribution ranging from 8 to 720 µm and heterogeneous surface characteristics. In contrast, controlled crystallization generated more uniform particles with reduced agglomeration and narrower particle size distributions. Among the evaluated methods, sonocrystallization provided the most effective control over particle size and morphology, demonstrated by a narrow size distribution ranging from 16 to 39 µm which correlated with improved flowability and surface energy. **Conclusions**: The study demonstrates that the choice of crystallization method significantly influences the structural and physicochemical properties of nicergoline. These findings highlight the importance of method selection for tailoring API properties to enhance downstream processing and product quality.

## 1. Introduction

Crystallization stands as one of the pharmaceutical industry’s most effective purification and separation methods. This process significantly impacts the physical characteristics of the final material, including particle size distribution, residual solvent levels, and overall purity [1,2,3,4,5]. Moreover, the method of crystallization influences both the surface and bulk properties of materials, which directly affect material handling and formulation processes. In pharmaceutical industry, four primary crystallization techniques are commonly employed: cooling, antisolvent addition, evaporative crystallization, and reactive crystallization, often in combination. These diverse methods not only impact pharmacopeial specifications, such as purity and residual solvent content, but also shape parameters like particle morphology and size distribution [6]. Additionally, the crystallization process exerts profound effects on surface and bulk properties, which subsequently manifest in mechanical characteristics and flow behavior of APIs [7,8,9]. These properties play a crucial role in downstream processes such as filtration duration, drying time, milling, micronization, and material handling, ultimately influencing the final formulation [5]. Although extensive literature exists on the correlation between crystallization methods and pharmacopeial specifications [10], the relationship between crystallization methods and the surface and rheological properties of APIs remains largely unexplored [4].

The fundamental process influencing crystallization is nucleation [11,12]. Nucleation represents the inception of a new phase within a supersaturated system, playing a pivotal role in determining the properties of the resulting crystalline material. Nucleation can occur through two mechanisms: primary heterogeneous nucleation and secondary nucleation. Primary heterogeneous nucleation involves a surface-induced or catalyzed process, occurring at sites such as crystallizer walls, stirrers, and thermometer wells. Conversely, secondary nucleation is induced by deliberately introducing crystalline material into the supersaturated solution, typically through seeding. While primary heterogeneous nucleation is commonly employed in industrial practices for API production, it often yields materials with heterogeneous properties due to its uncontrolled nature. In contrast, seeding-induced nucleation is a well-controlled process, resulting in a more uniform final product. During this process, nucleation and crystal growth occur simultaneously, with the quantity and particle size distribution of the seeding material determining the dominance of either process. Recent techniques, such as sonocrystallization [13,14] and template crystallization, which also induce nucleation, hold promise for enhancing control over the crystallization process.

Current trends in the pharmaceutical industry necessitate the synthesis of APIs with precise parameters (beyond pharmacopoeia), given, e.g., by the final formulation. These parameters encompass various factors, such as particle size and rheology, which significantly influence the performance and efficacy of the final product. Consequently, there is a growing demand for a comprehensive understanding of crystallization processes and the development of methodologies capable of producing crystals with tailored properties. This task presents a formidable challenge, particularly in the case of soft crystals formed by organic heterocyclic compounds, where achieving the desired characteristics requires innovative processes.

**Figure 1:**
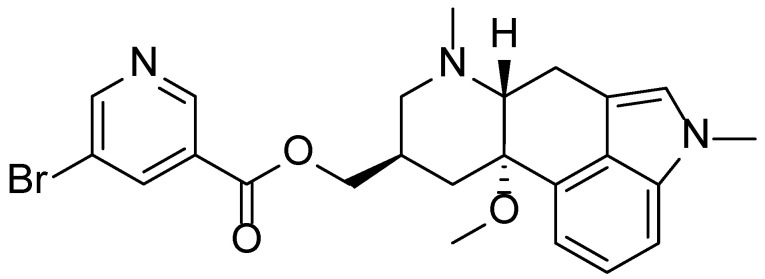
Chemical formula of nicergoline, [(6a*R*,9*R*,10a*S*)-10a-methoxy-4,7-dimethyl-6a,8,9,10-tetrahydro-6*H*-indolo[4,3-fg]quinolin-9-yl]methyl 5-bromopyridine-3-carboxylate.

Nicergoline, an ergot derivative with vasodilating activity, is a heterocyclic organic compound (Figure 1). Widely employed in human medicine, nicergoline is utilized for the treatment of cognitive disorders, including age-related cognitive impairment, as well as cerebrovascular disorders. Additionally, nicergoline has demonstrated efficiency in addressing hyperprolactinemia and Parkinson’s disease [15,16,17]. First introduced in 1972 by the Italian pharmaceutical company Farmitalia, which is now a part of Pfizer, nicergoline has a long-standing history in therapeutic applications. Teva Pharmaceutical Industries is recognized as a leading global producer of active substances, with an annual output amounting to several tons. Crystallization of nicergoline represents one of the final steps in the manufacturing of this API, which affects properties of final powder. Here, we focused on direct comparison of controlled crystallization techniques, such as seeding and sonocrystallization, with uncontrolled approaches, including cubic and linear cooling and acetone evaporation, to produce nicergoline. Beyond the pharmacopeial specifications emphasized in prior studies, our work demonstrates that crystallization methods influence both surface and bulk physicochemical properties, enabling the design of tailored API powder characteristics suited for industrial processing.

We employed various crystallization methods to produce nicergoline crystals and conducted a comprehensive analysis of the resulting crystalline powders. Our investigation centered on key parameters, including particle size distribution (PSD), specific surface area (SSA), surface energy (SE), and flow properties. SE, in particular, emerged as a potent tool for characterizing powder materials, offering insights into surface free energy, its components, surface heterogeneity, and other surface properties [18,19]. Notably, SE can be effectively assessed using inverse gas chromatography (IGC), as evidenced by numerous studies exploring the impact of different processing conditions [20,21,22], batch-to-batch variability [23,24,25], excipient–API interactions [26,27], and material heterogeneity [28,29]. SE analysis can also distinguish between the phases of a material, i.e., amorphous or crystalline [30,31,32]. Additionally, individual preparation techniques such as milling and micronization exert notable influences on the SE of the final powder material [20,33,34,35,36,37]. Thus, our focus was on elucidating the interrelationships among the properties of nicergoline powders obtained via different crystallization methods, aiming to exert precise control over the properties of the final product. Ultrasound-induced crystallization appears to be the optimal method for preparing nicergoline powder, significantly enhancing both particle properties and powder bulk behavior. Our findings indicate that a choice of crystallization method represents a cost-effective and easy-to-use industrial approach for preparing API powder with tailored rheological properties.

## 2. Results and Discussion

The batches of nicergoline under investigation were prepared by different crystallization techniques including cubic (CC) and linear cooling (LC), solvent evaporation (EC), induction by sonication (SC) and seeding (SLC) (see Table 1), which determined their physical–chemical properties. To elucidate the relationship between crystallization and the physical characteristics of the crystallized API, we characterized the prepared samples and analyzed their bulk and surface properties by a wide set of experimental methods.

### 2.1. Crystal Properties

**Figure 2:**
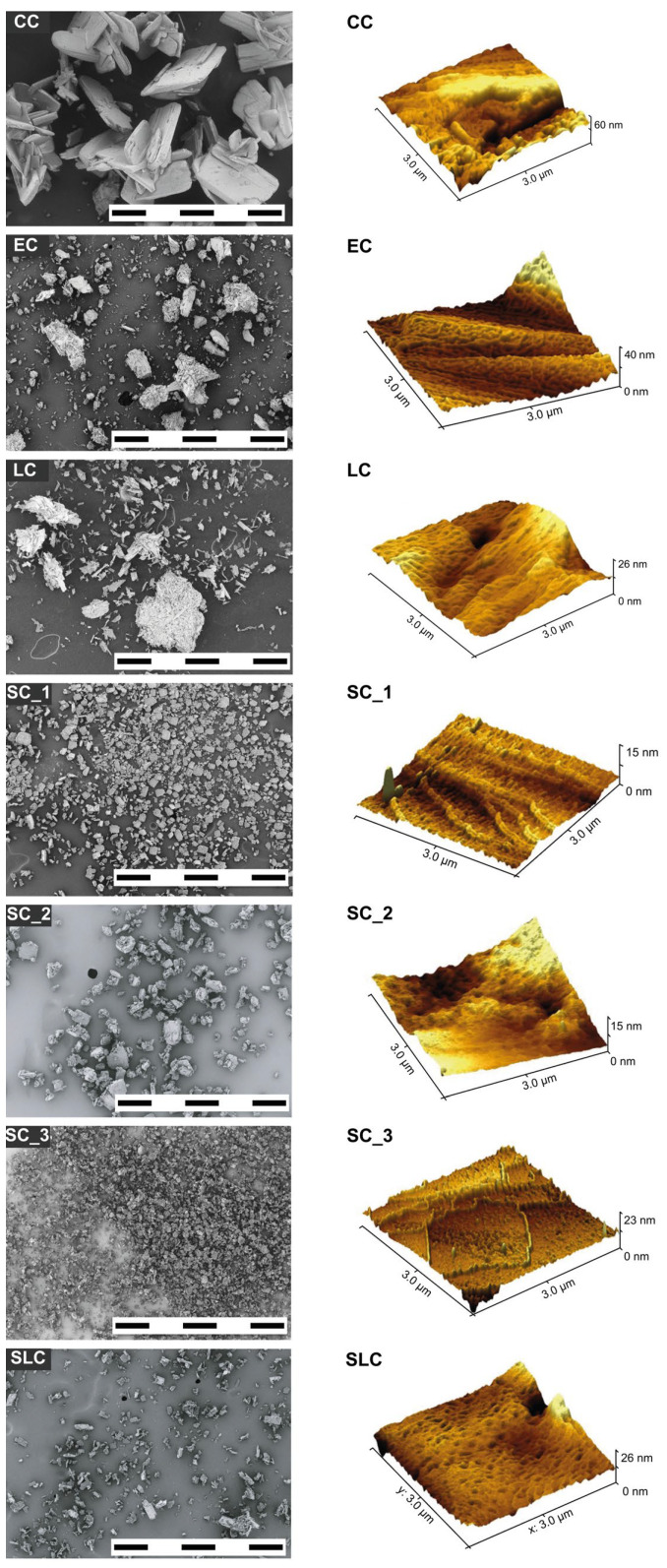
Images from scanning electron microscope (SEM) (**left** panels) and images from atomic force microscope (AFM) (**right** panels) of nicergoline prepared by different crystallization methods (see Table 1); top to bottom the nicergoline samples are CC (cubic cooling), EC (solvent evaporation), LC (linear cooling), SC_1, SC_2, SC_3 (sonication), and SLC (seeding). The scale bar for the SEM pictures is 1 μm devided in 5 segments of 200 nm each.

The nicergoline batches exhibit distinct crystal shapes, as observed via SEM analysis (Figure 2). Specifically, CC features flake crystals, EC displays acicular crystals, SLC presents equant crystals, LC showcases needle crystals, and SC demonstrates plate crystals (Figure 2). Notably, nicergoline batches crystallized via sonocrystallization (SC) exhibit the narrowest particle size distribution (Table 2), potentially attributed to the mechanical disruption of agglomerates or crystal disturbance during the sonocrystallization process [38], where the time of sonication affects the PSD. Conversely, nicergoline crystallized through acetone evaporation (EC) shows the widest particle size distribution, ranging from 8 µm (PSD (10)) to 720 µm (PSD (90)), with larger particles observed as agglomerates (Figure 2). Agglomerates tend to prolong drying times, highlighting the importance of minimizing their presence during crystallization to optimize dissolution properties [39]. Notably, CC exhibits the largest particles, with an average size of 107 µm (PSD (50)), while all batches from uncontrolled crystallization display wider particle size distributions and are more sensitive to agglomeration or re-agglomeration compared to controlled batches (Figure 2). Additionally, we assessed the particle/batch roughness via AFM analysis, revealing a range from 4.5 nm for CC to 0.6 nm for SC_1 (Table 2 and Figure 2). CC displays the highest roughness, likely attributed to its flaky crystals with cavities and edges, whereas SC_1 exhibits the lowest roughness, possibly due to the smoothing effect of sonocrystallization on crystal surfaces [40]. Consequently, batches prepared via controlled crystallization exhibit superior particle size distribution, reduced roughness, and lower susceptibility to agglomerate formation.

### 2.2. Residual Acetone

All samples met the pharmacopeial limit for residual acetone of 5000 ppm. SC_3 exhibits the lowest residual acetone content, approximately 590 ppm, along with three additional samples, SC_1, SC_2, and CC, which contain less than 1000 ppm of residual acetone. The smoother surface achieved through sonocrystallization reduces the likelihood of residual acetone being trapped in pores [40]. In contrast, LC contains the highest amount of residual acetone, approximately 4038 ppm, nearing the limit of 5000 ppm. Two more samples surpass the 1000 ppm threshold: EC with ~2440 ppm and SLC with ~1530 ppm (Table 2).

### 2.3. Surface Properties

The SSAs of the studied powders varied from 0.094 to 0.795 m^2^/g. Among them, CC exhibited the lowest specific surface area, while EC displayed the highest one (Table 2). Typically, surface area is expected to correlate with PSD, with decreased PSD leading to increased SSA. However, in our case, the significant differences are likely attributable to variations in size, shape, and porosity among the samples.

Detailed surface properties were probed by IGC. N-hexane, heptane, octane, and nonane were used as the probes for calculations of the dispersive part, while ethyl acetate and dichloromethane were used as acid-base probes. The measurement was carried out in the surface coverage of monolayer from 2 to 20%, except CC. CC was carried in the surface coverage from 5 to 20% due to a low surface area.

**Figure 3:**
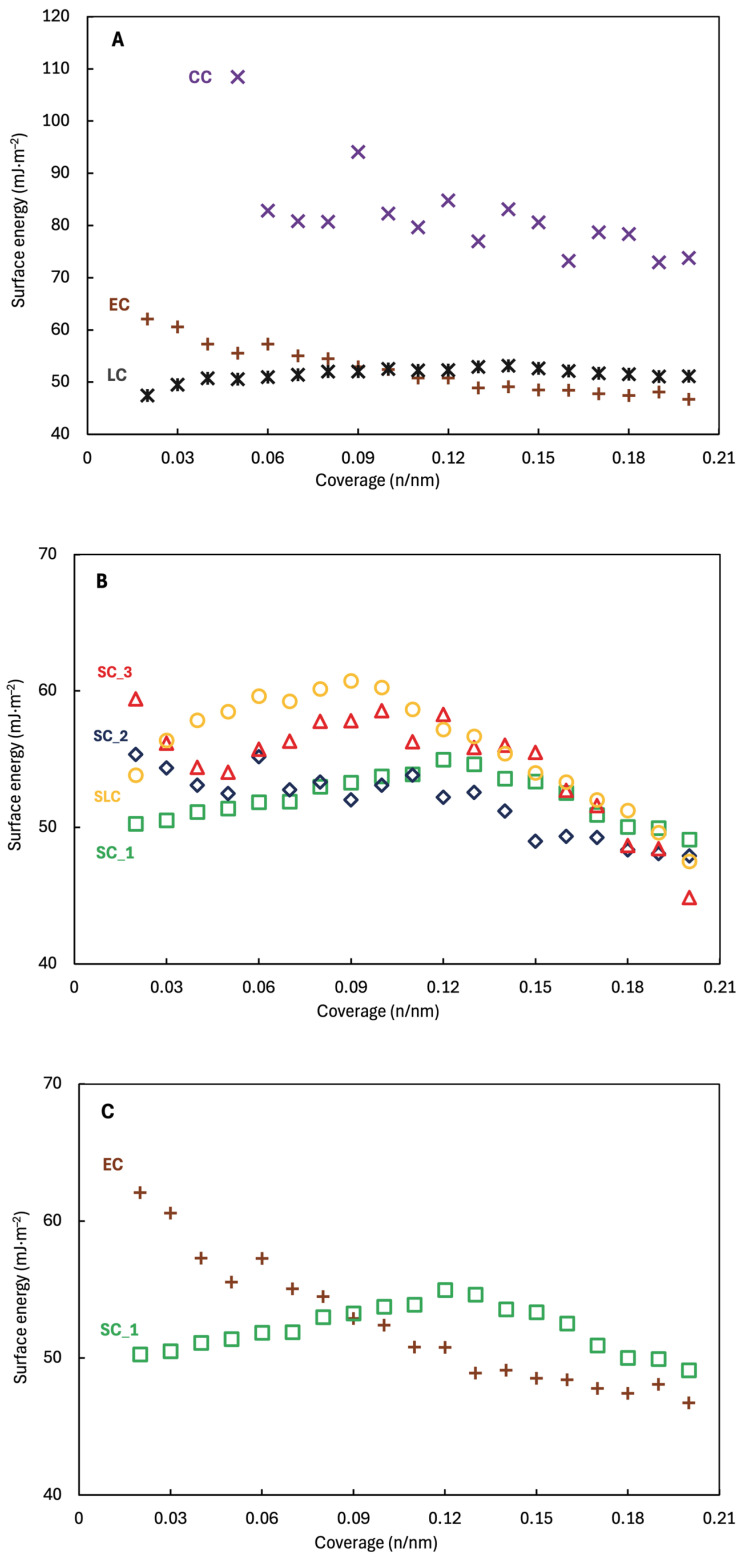
Total surface energy vs. coverage for samples prepared by (**A**) uncontrolled crystallization; (**B**) controlled crystallization; and (**C**) controlled (SC_1) vs. uncontrolled (EC).

The total surface energy of all nicergoline samples were dominated by the dispersive component, as the acid base contributions amount to ~ 4–6% of the total surface energy. The dominance of the dispersive component of the surface energy documents that the surfaces are of the non-polar nature. The differences among the surface energies of all samples are small (Figure 3). The highest surface energy has CC ~108.5 mJ/m^2^ followed by EC ~62.1 mJ/m^2^. On the contrary, the lowest surface energy has LC ~53.2 mJ/m^2^ followed by SC_1 ~55.0 mJ/m^2^. They also show the narrowest surface energy distribution LC ~5.8 mJ/m^2^, SC_1 ~5.9 mJ/m^2^, which is characteristic for energetically more homogeneous surfaces (Table 3). AFM measurement provided deep insights into these results, because the SC_1 displayed the lowest roughness, as it contains a minimum of cavities and edges. On the other hand, CC shows the widest surface energy distribution of ~35.6 mJ/m^2^, reflecting a higher surface heterogeneity. This batch should also be the most cohesive sample. Although EC is a relatively smooth sample, it has a wide range of particle size distribution. The cohesive behavior of those samples was also confirmed by flowability measurement.

**Figure 4:**
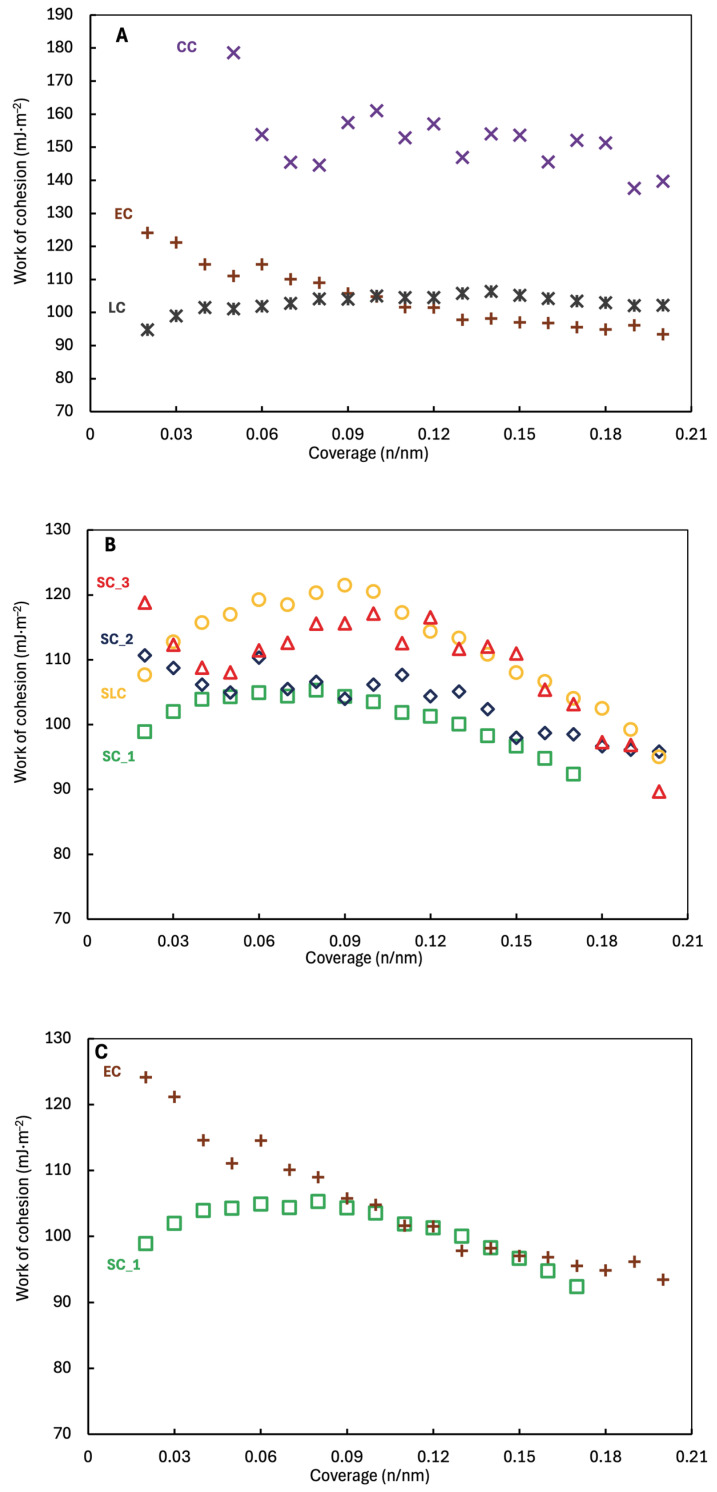
Work of cohesion vs. coverage of nicergoline samples prepared by (**A**) uncontrolled crystallization; (**B**) controlled crystallization; and (**C**) controlled (SC_1) vs. uncontrolled (EC).

**Figure 5:**
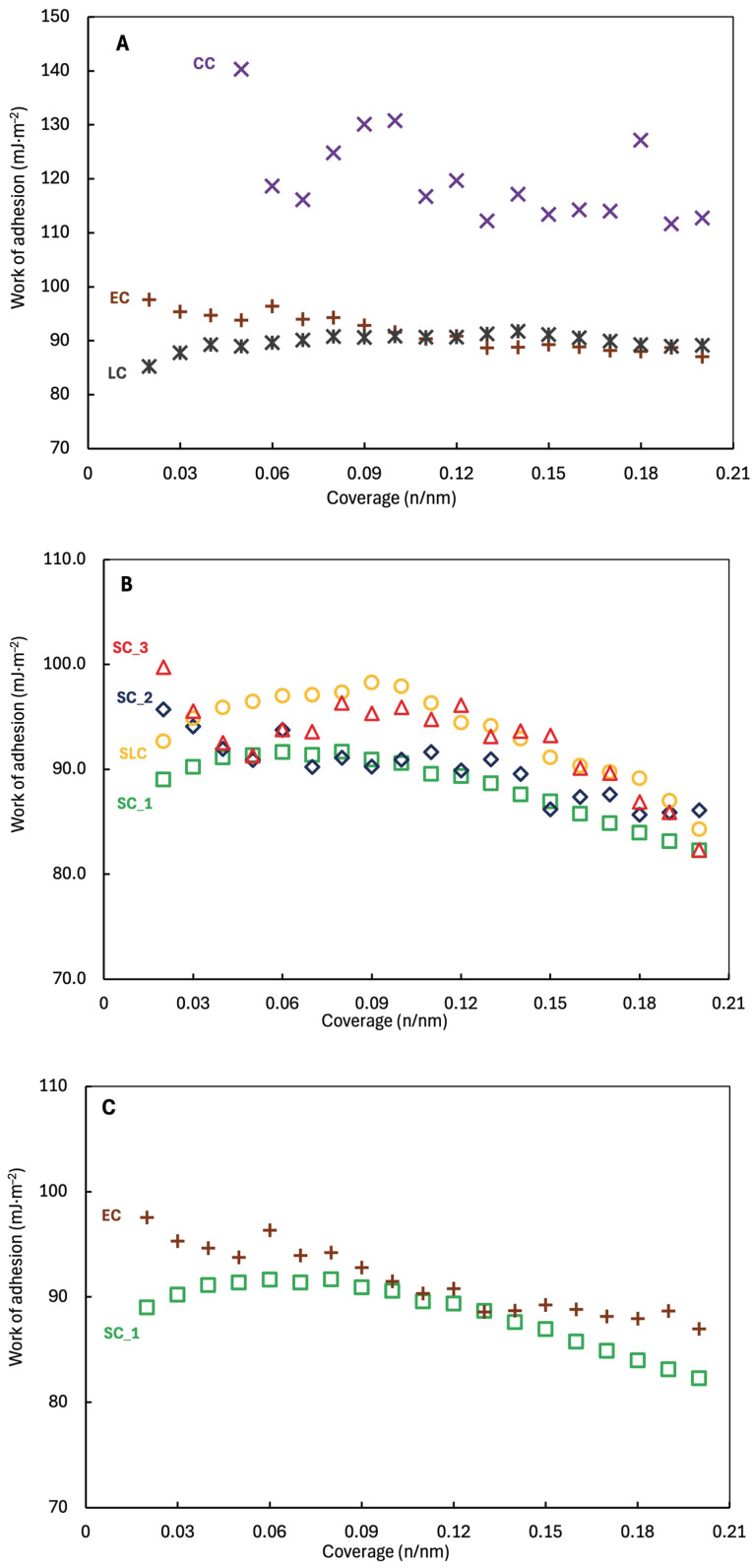
Work of adhesion vs. coverage of nicergoline samples prepared (**A**) uncontrolled crystallization; (**B**) controlled crystallization; and (**C**) controlled (SC_1) vs. uncontrolled (EC).

The difference between cohesive and adhesive work (Figure 4 and Figure 5) can tell us how the material will behave during formulation [41] and the strength of the interaction between APIs with carrier in the mixtures.

The particles interactions in the mixtures are further determined by the work of cohesion and adhesion [42]. The work of cohesion increases with stronger particle interaction, leading to greater agglomerates. However, strong adhesion may lead to stronger aggregation [43]. The nicergoline batches have similar profiles of total work of cohesion (Figure 4 and Figure 5). The highest work of cohesion has CC ~178.6 mJ/m^2^, as the lowest has SC_1 ~105.3 mJ/m^2^. Samples CC have the highest total work of adhesion (to water) ranging from ~140.2 mJ/m^2^ to ~111.6 mJ/m^2^. On the contrary, LC and SC_1 have the lowest total work of adhesion to water ranging from ~91.7 mJ/m^2^ (for both) to ~85.2 mJ/m^2^ or ~82.3 mJ/m^2^. All the data indicated that CC should be the most cohesive sample. The results again documented that the method of crystallization significantly affects the subsequent physicochemical properties, because of the way crystallization modules properties of crystals.

### 2.4. Powder Flow

The flow behavior of powders is primarily determined by two types of particle interactions: frictional forces and adhesive forces. These interactions primarily occur between particles themselves or between particles and contacting surfaces [44], often during various manufacturing processes such as grinding and mixing. We evaluated the flow function by examining its dependencies at different stress levels applied to the powder under study.

The measured *ff*s are presented in Table 2, indicating that SC_2, LC, and CC exhibit the most favorable flow properties. Specifically, SC_2 demonstrates a flowability of approximately 8.44, LC about 5.11, and CC around 4.44, suggesting characteristics typical of easily flowing powders. The SC_2 has relatively regular shape, which should decrease the friction and improve the flowability. Conversely, SLC is expected to possess the least desirable flow properties, as indicated by its flowability value of approximately 2.54, placing it in the range of very cohesive powders. The SLC has equant crystals that might be more prone to mechanical interlocking and thus resist to flow. At the same time, SLC has high roughness which increases friction and flow less easily. The remaining samples exhibit varying degrees of cohesion, with EC at approximately 3.27, SC_1 at about 3.63, and SC_3 at around 3.15. Increased cohesion in the material typically leads to challenges during subsequent handling processes.

## 3. Materials and Methods

Nicergoline (>99.5% pure), [(8β)-10-Methoxy-1,6-dimethylergolin-8-yl] methyl 5-bromopyridine-3-carboxylate, was supplied by TAPI Czech Industries s.r.o. (Opava, Czech Republic). Acetone (>99.0%) solvent for crystallization was supplied by Sigma Aldrich, Burlington, MA, USA; all solvents probes (n-hexane, heptane, octane, nonane, dichloromethane, and ethyl acetate) were of HPLC grade and were purchased from Sigma Aldrich.

Crystallization experiments were performed in Easy Max 102 (Mettler Toledo, Columbus, OH, USA) apparatus in 100 mL glass reactor (inner diameter 52 mm) with stainless steel anchor stirrer (outer diameter 48 mm). Active pharmaceutical substance nicergoline was crystallized in seven different experiments, keeping the same process parameters such as starting concentration (180 mg/mL), time and speed of the stirring, the filtration, and drying conditions. Individual samples were prepared by this way: 10 g of the substance was dissolved in 55 mL of acetone at the temperature 42–43 °C, the temperature was then kept 5 °C above the saturation temperature for 30 min in order to ensure complete dissolution. After this time the solution was cooled during 30 min to the temperature 40 °C. Experiment linear cooling (LC) was cooled by linear ramp 0.66 K·min^−1^ (from 40–0 °C), experiment crystallization induced by seeding (SLC) was seeded at 40 °C, and amount of the seed was 1% of the amount of the solute in solution and cooled the same temperature ramp as in previous experiment.

Experiment cubic cooling (CC) crystallization was cooled from 40 °C by cooling protocol according to Equation (1). Cooling profiles were programmed using iCare program (Mettler Toledo). Time of cooling was kept from 40 °C to 0 °C for 1 h. The used temperature profile can be described using the simplified equation:

(1) T=Tmax−Tmax− Tmin×tttotal3,

where *T* is the temperature at time *t*, *T_max_* and *T_min_* are the initial and final temperature at crystallization, and *t_total_* is the total crystallization time.

Experiment crystallization was produced by the evaporation of acetone (EC) from initial concentration 180 mg/mL to final concentration 360 mg/mL solute in solution at temperature 40 °C under reduced pressure. The nucleation in the last experiment, SC_1, was induced by sonication (SC), during which the ultrasound probe was installed in the center of the 100 mL glass reactor and immersed 20 mm in the solution. A magnetic stirrer (IKA) agitated the solution at speed 250 rpm. The sonication was performed in 750 W ultrasonic processor with the frequency of 20 KHz (Sonics & Materials, Inc., Newtown, CT, USA). The parameters of sonocrystallization were 40% amplitude and 2 s sonication and 2 s pause. Nicergoline crystallized out 2 min after sonication started and then it was again 1 h cooling ramp from 40 to 0 °C for 1 h. Experiment SC_2 was also induced by sonication with parameters 40% amplitude and 2 s sonication, 4 s pause. Nicergoline crystallized out after 5 min and then the suspension was sonicated for 15 min. Experiment SC_3 was sonicated with 40% amplitude and 4 s sonication and 2 s pause. Nicergoline crystallized out after cca 1 min and then the suspension was sonicated for 15 min. Sonocrystallization is mainly used in cases where the final crystalline material should exhibit a narrow particle size distribution with small particles. Sonocrystallization, as a technique involving the application of ultrasonic energy during the crystallization process, has demonstrated significant potential in controlling crystal properties of APIs. The cavitation phenomena induced by ultrasound promote nucleation and can lead to narrower particle size distributions, improved reproducibility, and enhanced control over crystal morphology. In this study, sonocrystallization was employed as a method to achieve targeted PSD, thereby aligning the final product characteristics with specific customer requirements. The results confirm that sonocrystallization is a robust and scalable approach for tailoring API crystallization outcomes.

All samples after cooling to 0 °C were stirred at this temperature for 60 min and then the crystalline material was filtrated through the filter (Sintr S3, Simax, Kavalierglass, Czech republic), washed with a small portion of cooled acetone, and dried at 50 °C for 5 h under a stream of nitrogen.

We characterized the surface and bulk properties of the crystallized samples (see Table 1 with an overview of used crystallization methods) using a wide range of physical–chemical methods. Particle size distribution of nicergoline was measured by laser diffraction using the instrument Malvern Mastersizer 2000 (Malvern Instruments, Malvern, UK); the samples were diluted by water as dispersion medium with 1% Tween 80. The amount of residual acetone was measured by headspace gas chromatography (7697+7890, Agilent Technologies, Santa Clara, CA, USA) using a FID (flame ionization detector) and special capillary column for fast determination of RS with USP<467> phase G43 (6% cyanopropyl phenyl/ 94% methyl polysiloxane). Scanning electron microscope (SEM) (Hitachi TM3030 Plus; Hitachi, Tokyo, Japan) with cathode EM, electron beam energy of 5kV was used to monitor shape and size of the samples. Samples were applied on alumina target in flow box and coated with a thin layer of metal (Au). Target was covered with double-faced carbon adhesive tape. Prepared sample was placed into the scanning electron microscope and evacuated. Atomic force microscope (AFM) (NTEGRA Spectra SOLAR, Shanghai, China) was applied to measure the roughness of the surface. HA_NC (etalon) probe, with resonance frequency of 140 kHZ, was utilized for all measurements. Samples were applied on the adhesive tape located on the sample mounting disk. The surface of the samples was scanned in 7 different areas; each area was 3 × 3 µm^2^. The specific surface area was measured by Autosorb iQ (Qantachrome Instruments, Boynton Beach, FL, USA) using krypton as the adsorbed gas. The 0.4 g powder samples were filled into 9 mm (internal diameter) cells with glass filer rod. The samples were degassed for 12 h at 25 °C in an outgas station, to release off gas or vapor from the powder sample. The sample was evacuated and tempered to −196 °C by immersion into a cold trap dewar flask with liquid nitrogen. Helium was used as a void volume mode and krypton as the analysis gas.

IGC was carried out using a Surface Energy Analyzer instrument (Surface Measurement Systems, Wembley, UK). The 1 g powder samples were packed into a silanized glass column (30 cm long with 4 mm internal diameter). Ends of the column were filled with glass wool. The filled column was mounted into a column sample oven (30 °C). N-hexane, heptane, octane, and nonane were used as non-polar solvents and dichloromethane and ethyl acetate were used as polar solvent probes. Methane gas was the reference probe. The injection of vapors was regulated to pass an adjusted volume of eluent through the column to obtain pre-destined fractional coverage of the sample in the column. The eluted vapors were detected by flame ionization detectors. The dispersive, acid-base, and total surface energy of nicergoline samples were acquired from the SEA Cirrus Plus software (version 1.4.1.0).

The dispersive surface energy was determined using Doris and Gray method [45,46], Equation (2)

(2) $$\gamma_{S}^{D}=\frac{\left[RT\ln\left(V_{N\left(C_{n+1}H_{2n+4}\right)}\right)/V_{N\left(C_{n}H_{2n+2}\right)}\right]}{4N_{A}^{2}a_{CH_{2}}^{2}\gamma_{CH_{2}}},$$

where $a_{{CH}_{2}}$ is the surface area of a CH_2_ unit (~0.06 nm^2^) and $\gamma_{CH_{2}}$ is the free energy of CH_2_ (approximately 35.6 mJ/m^2^). The${\;\gamma }_{S}^{D}\;$is calculated from the slope of line in a plot of $RTlnV_{N}$ vs. $a{\left(\gamma_{L}^{D}\right)}^{1/2}$. Della Volpe scale was used to obtain the acid-base part of surface energy [47]. The total surface energy was calculated as a sum of dispersive and acid-base parts.

Work of cohesion was calculated from equation

(3) $$W_{coh}=W_{coh}^{d}+W_{coh}^{ab},$$

(4) $$W_{coh}^{d}=2{\left(\gamma_{s}^{d}\gamma_{s}^{d}\right)}^{1/2},$$

(5) $$W_{coh}^{ab}=2{\left(\gamma_{s}^{+}\gamma_{s}^{-}\right)}^{1/2}+{\left(\gamma_{s}^{-}\gamma_{s}^{+}\right)}^{1/2},$$

where $\gamma_{s}^{d}$ is the dispersive component of the surface energy of solid.

Work of adhesion is defined as

(6) $$W_{adh}=2{\left(\gamma_{s}^{d}\gamma_{l}^{d}\right)}^{1/2},$$

(7) $$W_{adh}^{ab}=2{\left(\gamma_{s}^{+}\gamma_{l}^{-}\right)}^{1/2}+{\left(\gamma_{s}^{-}\gamma_{l}^{+}\right)}^{1/2},$$

where $\gamma_{s}^{d}$ is the dispersive component of the surface energy of solid and $\gamma_{l}^{d}$ is the dispersive component of the surface energy of the probe molecule. Water was used as the probe molecule.

Flowability was conducted on the FT4 Powder Rheometer (Freeman Technology, Tewkesbury, UK) using the shear test measurement. The shear test was applied on the 1.5–2.0 g loaded powder. The flow properties were determined from the Mohr stress circles using the FT4 data analysis. The measured flow factor (*ff*), as defined by Jenike [44], Equation (8), is characterized by the ratio of the unconfined yield strength $\sigma_{C}$, to the consolidation stress, *σ*_1_:

(8) $$ff=\;\frac{\sigma_{1}}{\sigma_{C}}=\frac{MPS}{UYS},$$

$UYS/\sigma_{C}$ (unconfined yield stress, kPa) is the maximum normal stress value, when free and stress surface flows less or deforms. $MPS/\sigma_{1}$ (major principal stress, kPa) is received from Mohr stress circle of the steady-state flow for an applied normal consolidation stress. $MCS/\sigma_{2}$ (minor consolidation stress, kPa) is the minor principal stress acting on the powder. The flow factor coefficient is determined using 9 kPa pre-consolidation stress and is derived from measurements obtain using FT4 rheometer. The flow factor classifies the powder flow according to Jenike as follows: *ff* < 1 not flowing powder, 1 < *ff* < 2 very cohesive, 2 < *ff* < 4 cohesive, 4 < *ff* < 10 easy flowing, and *ff* > 10 free flowing powder.

## 4. Conclusions

Crystallization methods play a significant role in shaping the surface and volume properties of the final product. The nicergoline products prepared by controlled crystallization show more uniform behavior with desired final parameters. The controlled crystallization narrows the PSD and improves the levels of residuals. The controlled crystallization also prevents the formation of agglomerates. On the other hand, the uncontrolled crystallization tends to produce powders with wider PSD and higher residual solvent levels. The sonocrystallization yields smoother surfaces, narrower PSD, and fewer agglomerates. Additionally, ultrasound treatment results in reduced residual solvent content. Conversely, controlled nucleation, particularly, enables us to generate samples with the desired properties, potentially minimizing the need for extensive post-processing typically employed to achieve the desired characteristics of an API powder. Generally, the controlled nucleation provides a practical route to obtain products with well-defined properties, which may lessen the need for extensive post-processing and support the development of more efficient strategies for API powder design.

## Figures and Tables

**Table 1:** Experimental crystallization methods and setup conditions used in this study.

Label	Type of Crystallization	Controlled	Uncontrolled	Sonification
CC	Cubic cooling		X	-
EC	Evaporation of acetone		X	-
LC	Linear cooling		X	-
SC_1	Induced by sonification	X		40% amplitude, 2 s. sonication, 2 s. pause
SC_2		X		40% amplitude, 2 s. sonication, 4 s. pause
SC_3		X		40% amplitude, 4 s. sonication, 2 s. pause
SLC	Induced by seeding	X		-

**Table 2:** Amount of residual acetone GC [ppm], particle size distribution [µm], root mean square [nm], specific surface area [m^2^/g] and flow factor (*ff*) of microcrystal powders prepared by five different crystallization techniques.

Sample	GC [ppm]	PSD (10) [µm]	PSD (50) [µm]	PSD (90) [µm]	RMS [nm]	RMS [nm] Range	SSA [m^2^/g]	*ff*
CC	699	43	107	218	4.5 ± 3.7	1.6 … 13.3	0.094	4.44
EC	2440	8	80	720	1.8 ± 1.0	0.6 … 3.4	0.795	3.27
LC	4038	5	28	87	1.2 ± 0.8	0.5 … 2.9	0.481	5.11
SC_1	705	12	31	60	0.6 ± 0.1	0.1 … 0.8	0.401	3.63
SC_2	820	9	33	81	1.3 ± 0.4	0.7 … 1.9	0.262	8.44
SC_3	590	5	16	39	1.0 ± 0.3	0.7 … 1.6	0.582	3.15
SLC	1530	5	33	75	3.6 ± 1.8	0.9 … 6.5	0.666	2.54

**Table 3:** Total surface energy γ_t_ (their high and low values), total work of cohesion Wcoh_t_, and total work of adhesion Wadh_t_ [mJ/m^2^].

Sample		γ_t_ [mJ/m^2^]	Δγ_t_ [mJ/m^2^]	Wcoh_t_ [mJ/m^2^]	Wadh_t_ [mJ/m^2^]
CC	max	108.5	35.6	178.6	140.2
	min	72.9		137.6	111.6
EC	max	62.1	15.4	124.2	97.6
	min	46.7		93.5	87.0
LC	max	53.2	5.8	106.3	91.7
	min	47.4		94.8	85.2
SC_1	max	55.0	5.9	105.3	91.7
	min	49.1		87.4	82.3
SC_2	max	55.3	7.4	110.7	95.7
	min	47.9		95.8	85.7
SC_3	max	59.4	14.6	118.8	99.7
	min	44.8		89.7	82.3
SLC	max	60.7	13.2	121.5	98.3
	min	47.5		95.0	84.3

## Data Availability

Data will be available at ZENODO (https://doi.org/10.5281/zenodo.17219366).

## Abbreviations

The following abbreviations are used in this manuscript:

API	active pharmaceutical ingredient
PSD	particle size distribution
SSA	specific surface area
SE	surface energy
IGC	inverse gas chromatography
CC	cubic cooling
LC	linear cooling
EC	solvent evaporation (evaporation of acetone)
SC	induction by sonication
SLC	induction by seeding
*ff*	flow factor
GC	gas chromatography
RMS	root mean square
SEM	scanning electron microscope
AFM	atomic force microscope
*γ_t_*	total surface energy
Wcoh	work of cohesion
Wadh	work of adhesion
HPLC	high-performance liquid chromatography
*T*	temperature
*t*	time
FID	flame ionization detector
RS	residual solvents
USP	United States pharmacopeia
EM	electron beam
HA_NC	brand name for AFM probe
SEA	surface energy analyzer
